# Ascorbic Acid Has Superior Ex Vivo Antiproliferative, Cell Death-Inducing and Immunomodulatory Effects over IFN-α in HTLV-1-Associated Myelopathy

**DOI:** 10.1371/journal.pntd.0001729

**Published:** 2012-07-24

**Authors:** Britta Moens, Daniele Decanine, Soraya Maria Menezes, Ricardo Khouri, Gilvanéia Silva-Santos, Giovanni Lopez, Carolina Alvarez, Michael Talledo, Eduardo Gotuzzo, Ramon de Almeida Kruschewsky, Bernardo Galvão-Castro, Anne-Mieke Vandamme, Johan Van Weyenbergh

**Affiliations:** 1 Rega Institute for Medical Research, K. U. Leuven, Leuven, Belgium; 2 Gonçalo Moniz Research Center, Oswaldo Cruz Foundation (FIOCRUZ), Salvador-Bahia, Brazil; 3 Instituto de Medicina Tropical Alexander von Humboldt, Universidad Peruana Cayetano Heredia, Lima, Peru; 4 Departamento de Medicina, Facultad de Medicina, Universidad Peruana Cayetano Heredia, Lima, Peru; 5 Bahia School of Medicine and Public Health, Salvador-Bahia, Brazil; 6 Centro de Malária e outras Doenças Tropicais, Instituto de Higiene e Medicina Tropical, Universidade Nova de Lisboa, Lisboa, Portugal; Hospital Universitário, Brazil

## Abstract

**Background:**

Clear therapeutic guidelines for HTLV-1-associated myelopathy/tropical spastic paraparesis (HAM/TSP) are missing due to the lack of randomized double-blind controlled clinical trials. Moderate yet similar clinical benefit has been demonstrated for IFN-α and high-dose ascorbic acid (AA) monotherapy in a large open clinical trial. However, there is a lack of *in vivo* and *in vitro* studies exploring and comparing the effects of high-dose AA and IFN-α treatment in the context of HAM/TSP. Therefore, we performed the first comparative analysis of the *ex vivo* and *in vitro* molecular and cellular mechanisms of action of IFN-α and high-dose AA in HAM/TSP.

**Principal Findings:**

Through thymidine incorporation and quantification of Th1/Th2/Th17 cytokines, we demonstrate that high-dose AA displays differential and superior antiproliferative and immunomodulatory effects over IFN-α in HAM/TSP PBMCs *ex vivo*. In addition, high-dose AA, but not IFN-α, induced cell death in both HAM/TSP PBMCs and HTLV-1-infected T-cell lines MT-2 and MT-4. Microarray data combined with pathway analysis of MT-2 cells revealed AA-induced regulation of genes associated with cell death, including *miR-155*. Since miR-155 has recently been demonstrated to up-regulate IFN-γ, this microRNA might represent a novel therapeutic target in HAM/TSP, as recently demonstrated in multiple sclerosis, another neuroinflammatory disease. On the other hand, IFN-α selectively up-regulated antiviral and immune-related genes.

**Conclusions:**

In comparison to IFN-α, high-dose AA treatment has superior *ex vivo* and *in vitro* cell death-inducing, antiproliferative and immunomodulatory anti-HTLV-1 effects. Differential pathway activation by both drugs opens up avenues for targeted treatment in specific patient subsets.

## Author Summary

HAM/TSP is a chronic and disabling neuroinflammatory disease, for which clinical management is mostly empirical and symptomatic rather than evidence-based, due to the lack of biomarkers and controlled clinical trials. Although similar clinical benefit has been demonstrated for IFN-α and high-dose ascorbic acid (vitamin C) in one major open clinical trial with 200 patients, their cellular and molecular mechanisms of action remain unexplored in HAM/TSP. We demonstrate that high-dose ascorbic acid strongly inhibits lymphoproliferation of HAM/TSP mononuclear cells in *ex vivo* cultures, in contrast to IFN-α. Furthermore, high-dose ascorbic acid, but not IFN-α, significantly decreased *ex vivo* TNF-α and IFN-γ pro-inflammatory cytokine levels in supernatant of mononuclear cells from HAM/TSP patients. In addition, ascorbic acid, but not IFN-α, induced cell death in HTLV-1-infected T-cell lines, which was confirmed by gene expression profiling, revealing cell death-associated pathways activated by high-dose ascorbic acid, including *miR-155*. This microRNA has previously been shown up-regulated in HTLV-1-infected cells, as well as in blood and brain samples of multiple sclerosis patients, another neuroinflammatory disease. In addition, miR-155 has also been reported to up-regulate IFN-γ production in human natural killer cells, thus linking both cell death and cytokine signaling pathways, rendering it a potential therapeutic target in neuroinflammatory disorders. Thus, our findings reveal molecular mechanisms of action as well as candidate biomarkers for high-dose ascorbic acid therapy and provide a rational basis, rather than an empirical basis, for its use in HAM/TSP treatment.

## Introduction

In human T-lymphotropic virus type 1 (HTLV-1) infection, approximately 0.3–4% of infected individuals develop a progressive neurodegenerative disease termed HAM/TSP or HTLV-1-associated myelopathy/tropical spastic paraparesis [1], whereas the majority of infected individuals remain clinically asymptomatic throughout their lifetimes. HAM/TSP is characterized by demyelinating lesions in the central nervous system (CNS), mainly located in the thoracic and lumbar spinal cord regions. Pathogenesis remains poorly understood and attention has been particularly focused on the cellular and humoral immune response to HTLV-1 infection [2], [3]. It is assumed that the HTLV-1-specific CD8+ cytotoxic T cell (CTL) response may lead to bystander neural tissue damage when recruited in the CNS through release of proinflammatory cytokines [4]. The most prominent symptoms of HAM/TSP patients are weakness, spasticity and hyperreflexia of the lower extremity, however lower back pain, bladder disturbance and sensory dysfunction are also frequently reported [5]. Disability and motor dysfunction reflecting severity of clinical symptoms, can be graded according to the expanded disability status scale (EDSS) or the Osame's motor disability score (OMDS).

At present, HAM/TSP treatment is mainly symptomatic and empirical. Clinical benefit has been demonstrated mainly for corticosteroids, antispasmodics, interferon-α and high-dose ascorbic acid (AA) treatment [6]–[8]. Oral administration of prednisolone seems to be the most effective treatment option, with a success rate of 69.5%, comprising gain of 1 grade or more in OMDS. Treatment with antispasmodics improved patient's outcome with 50%, followed by intramuscular IFN-α and oral high-dose AA, with a moderate success rate of 21.9% and 20%, respectively. However, no long-term clinical improvements are achieved using the above-mentioned therapies and there is no clear evidence to support the value of one of these specific treatment approaches over others due to the lack of randomized double-blind controlled clinical trials [8]. Therapeutic benefit has only been verified for IFN-α, based on a single double-blind randomized controlled clinical trial [6]. Considering the similar clinical benefit reported for IFN-α and high-dose AA treatment, the latter, due to its milder *in vivo* side effects and lower cost, is an attractive therapeutic alternative in neglected diseases such as HAM/TSP. AA is an essential nutrient acting as an antioxidant and co-factor for various enzymes [9]. Both immunomodulatory as well as antiproliferative effects have been described for AA, although controversy still exists [10]–[13]. In parallel, IFN-α has been reported to exert antiviral, immunomodulatory and antiproliferative effects in several types of human cancer and viral infections [14]–[16]. In contrast, *in vitro* studies exploring the potential effects of AA and IFN-α in the context of HAM/TSP are limited, although antiproliferative effects have been described for high-dose AA in HTLV-1-infected cell lines [17].

In the present study, we evaluated the *ex vivo* and *in vitro* effects of AA and IFN-α treatment on peripheral blood mononuclear cells (PBMCs) of seronegative normal donors, HTLV-1-infected asymptomatic carriers and HAM/TSP patients and HTLV-1-infected cell lines, respectively. We demonstrate superior antiproliferative, cell death-inducing and immunomodulatory effects of high-dose AA compared to IFN-α treatment, which are confirmed by microarray and pathway analysis.

## Methods

### Reagents

IFN-α2A (3×10^6^ IU/ml, a gift of Blausiegel Farmacêutica, São Paulo, Brazil) and ascorbic acid (AA, Sigma-Aldrich, Belgium) stock solutions were prepared in normal saline and milli-Q water, respectively. *N*-acetylcysteine (NAC, Sigma-Aldrich, Belgium) stock solutions were prepared in milli-Q water. Working solutions were prepared in RPMI 1640 medium, supplemented with 10% heat inactivated foetal calf serum, 20 µg/ml gentamicin and 75 mM NaHCO_3_ (GIBCO® Invitrogen, Belgium).

### Patients samples and cell lines

Diagnosis of HTLV-1 infection and HAM/TSP was made according to published criteria, hereby combining ELISA (Murex), Western blot, INNO-LIA (Innogenetics) and clinical data. Written informed consent was obtained from all participants and this study was approved by the Ethics Committee of CpqGM-FIOCRUZ and HUPES/UFBA (Salvador-Bahia, Brazil) and the Universidad Peruana Cayetano Heredia (Lima, Peru). PBMCs of ten normal donors (NDs), five asymptomatic carriers (ACs) and sixteen HAM/TSP patients were isolated by Ficoll-Hypaque density gradient centrifugation (Sigma-Aldrich). HAM/TSP patients were withdrawn from therapy 24 hours before blood sampling. Patient information regarding country of origin, EDSS, proviral load, treatment, age and gender, is listed in Table 1.

**Table 1:** Schematic overview of normal donors, asymptomatic carriers and HAM/TSP patients.

Number	Origin	EDSS	PVL	Therapy	Age	Gender
ND 1	Lima, Peru	0	/	/	26	F
ND 2	Lima, Peru	0	/	/	ND	F
ND 3	Lima, Peru	0	/	/	ND	F
ND 4	Lima, Peru	0	/	/	ND	F
ND 5	Bahia, Brazil	0	/	/	44	M
ND 6	Bahia, Brazil	0	/	/	31	F
ND 7	Bahia, Brazil	0	/	/	36	F
ND 8	Bahia, Brazil	0	/	/	ND	ND
ND 9	Bahia, Brazil	0	/	/	ND	ND
ND 10	Bahia, Brazil	0	/	/	ND	ND
AC 1	Lima, Peru	0	<10	/	46	M
AC 2	Lima, Peru	0	11	/	39	M
AC 3	Bahia, Brazil	0	ND	/	ND	ND
AC 4	Bahia, Brazil	0	ND	/	73	F
AC 5	Bahia, Brazil	0	ND	/	21	F
HAM/TSP 1	Lima, Peru	7	3024	predn+3TC+baclo	27	F
HAM/TSP 2	Lima, Peru	5	311	baclo	49	F
HAM/TSP 3	Lima, Peru	4.5	2782	predn+3TC	35	F
HAM/TSP 4	Lima, Peru	4	3805	baclo	50	F
HAM/TSP 5	Lima, Peru	6	2940	baclo+AZT	63	F
HAM/TSP 6	Lima, Peru	ND	3244	baclo	64	M
HAM/TSP 7	Lima, Peru	4	2599	baclo	64	F
HAM/TSP 8	Lima, Peru	6.5	3080	predn+3TC+baclo	32	F
HAM/TSP 9	Lima, Peru	6	ND	predn+3TC+baclo	48	F
HAM/TSP 10	Lima, Peru	ND	ND	baclo	42	F
HAM/TSP 11	Bahia, Brazil	5	ND	predn+AA	51	F
HAM/TSP 12	Bahia, Brazil	4	ND	predn+AA	59	M
HAM/TSP 13	Bahia, Brazil	5	ND	predn+AA	60	F
HAM/TSP 14	Bahia, Brazil	6	ND	untreated	56	M
HAM/TSP 15	Bahia, Brazil	4	ND	untreated	32	F
HAM/TSP 16	Bahia, Brazil	6	ND	untreated	53	F

EDSS = expanded disability status scale, “0” reflecting normal neurological examination and “10” death; PVL = proviral load expressed as HTLV-1 copies per 10^4^ PBMCs; ND = not determined; predn = prednisone; AA = high-dose ascorbic acid; 3TC = lamivudine; baclo = baclofen; AZT = zidovudine; F = female; M = male.

PBMCs, HTLV-1-infected T cell lines MT-2 and MT-4 [18], [19], and the uninfected Jurkat T cell line were cultured in RPMI 1640 medium supplemented with 10% heat inactivated foetal calf serum, 20 µg/ml gentamicin and 75 mM NaHCO_3_. For varying time spans, PBMCs and cell lines were cultured at 1×10^6^ cells/ml and 2×10^5^ cells/ml, respectively, in the absence or presence of AA (10, 50 or 100 µg/ml) or IFN-α2A (1000 IU/ml).

### Proliferation assays: thymidine incorporation and cell counting

Lymphoproliferation in PBMCs (1×10^6^ cells/ml) of NDs (n = 4) and HAM/TSP patients (n = 3) was quantified by [^3^H]thymidine (0.5 Ci/200 µl) incorporation after 4 days of treatment in the absence or presence of low-dose AA (10 µg/ml), high-dose AA (100 µg/ml), IFN-α2A (1000 IU/ml) and/or anti-CD3 monoclonal antibody (0.2 µg/ml) as a positive control. After overnight incubation (16 hours), [^3^H]thymidine uptake into DNA was measured using a LKB-β scintillation counter (Packard-Matrix™ 9600) and expressed as counts per minute (cpm). In addition, direct cell counts of viable and dead cells of NDs PBMCs (n = 3) and HAM/TSP PBMCs (n = 6) were quantified by trypan blue dye exclusion and microscopy after 72 hours of treatment in the absence or presence of low-dose AA (10 µg/ml), high-dose AA (100 µg/ml) and IFN-α2A (1000 IU/ml).

### Cytokine and HTLV-1 p19 detection

Th1/Th2/Th17 cytokines (interleukin-2 (IL-2), IL-4, IL-6, IL-10, tumor necrosis factor-alpha (TNF-α), interferon-gamma (IFN-γ) and IL-17) were quantified in cell-free supernatant of PBMCs of NDs (n = 7), ACs (n = 5) and HAM/TSP patients (n = 9) at 24–48–72 hours of treatment, using cytometric bead array kit (BD Biosciences). In addition, Th1/Th2/Th17 cytokines were also quantified in cell-free supernatant of HTLV-1-infected cell lines after 48 hours of treatment.

After 48 hours of treatment, HTLV-1 matrix protein p19 was quantified in cell-free supernatant of PBMCs of NDs (n = 7), ACs (n = 5) and HAM/TSP patients (n = 7) and in cell-free supernatant of HTLV-1-infected cell lines, using RetroTek HTLV-I/II p19 Antigen ELISA kit (ZeptoMetrix).

Both PBMCs and cell lines were treated in the absence or presence of high-dose AA (100 µg/ml) or IFN-α2A (1000 IU/ml).

### Flow cytometry assay

Flow cytometric quantification of DNA content (Hoechst 33342), proliferation-associated (PCNA) and cell death-associated markers (DNA degradation, active-caspase 3) was performed using fluorescence-labelled monoclonal antibodies and FACSCanto II (BD Biosciences). Briefly, cells were fixed in Cytofix buffer (BD Biosciences) for 10 minutes at 37°C and cell pellets were permeabilized in 100% ice-cold methanol for 30 minutes. Cells were then washed twice in 1x PBA (phosphate-buffered saline+bovine serum albumin+NaN_3_) and incubated with fluorescence-labelled monoclonal antibodies at room temperature (FITC mouse IgG2a, PE mouse IgG2a, FITC anti-PCNA, PE anti-active Caspase-3). After 30 minutes, cells were washed twice in 1x PBA and stained with Hoechst 33342. Cell populations and debris were defined based on morphology via forward-*versus* side-scatter plots and 10,000–100,000 events were acquired per sample.

### Microarray analysis

Total RNA was extracted from MT-2 cells treated for 48 hours in the absence or presence of AA (10, 50 or 100 µg/ml) or IFN-α (1000 IU/ml), using RNeasy kit according to the manufacturer's protocol (QIAgen Benelux B.V., VENLO, the Netherlands). Whole Human Genome microarray analysis was performed by the VIB MicroArray Facility (Leuven, Belgium). Data were analysed using the Affymetrix GeneChip software based on the Robust Multichip Average (RMA) expression values as obtained with the xps package version 1.8.0. The contrasts in expression between IFN-α, the three different doses of AA (low, intermediate, high) and no treatment at 48 hours of stimulation, were estimated using the Limma package from Bioconductor (www.bioconductor.org). For the selection of differentially transcribed genes, an uncorrected p-value cut off of p<0.001 was used.

Details on the construction of this microarray are available at NCBI (GEO Accession Number GSE34572).

### Ingenuity pathway analysis

The Ingenuity Pathway Analysis (IPA) program was used to perform a pathway/function level analysis on genes resulting from the microarray analysis on MT-2 cells (IPA version 9.0, Build 116623, Content version 3211, Ingenuity Systems, Red Wood City, CA). To have sufficient genes as input for the analysis (between 100 and 800 genes), uncorrected p-values were used with a cut-off of p<0.005, without using a cut-off on fold-change. Based on a scientific literature database, genes were sorted into gene networks and canonical pathways and significantly overrepresented pathways were identified (www.ingenuity.com). The maximum number of networks to be generated was set to 25, with a maximum number of 35 molecules per network.

### Statistical analysis

Statistical analysis was performed with GraphPad Prism 5 software. ANOVA (with Bonferroni post-test for multiple testing or post-test for linear trend, where appropriate) and t-test were used for parametric data (data shown with standard error of the mean), whereas Friedman test was used for non-parametric data. All tests were two-sided and p<0.05 was considered significant. Fisher's exact test was used for categorical data.

For microarray analysis, a moderated t-test was used, as implemented in the Limma package, to test whether a contrast was significantly different from zero.

## Results

### High-dose AA has superior antiproliferative effects over IFN-α in HAM/TSP PBMCs

**Figure 1:**
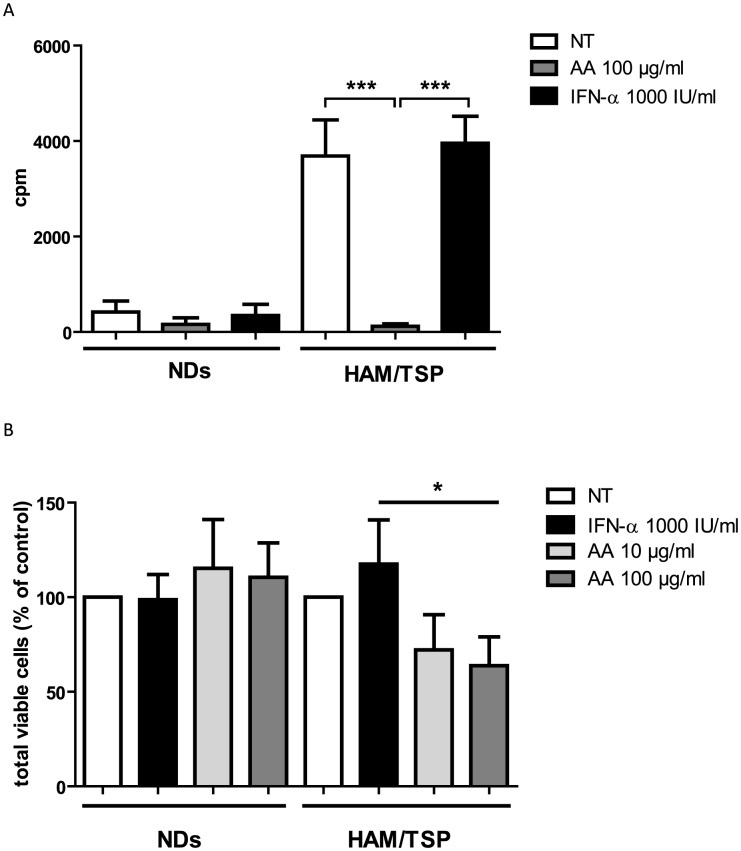
High-dose AA, but not IFN-α, exerts strong antiproliferative effects in HAM/TSP PBMCs. (A) Lymphoproliferation of normal donors (NDs) PBMCs (n = 4) and HAM/TSP PBMCs (n = 3) was quantified by [^3^H]thymidine incorporation (cpm), performed in triplicate. NDs and HAM/TSP PBMCs were cultured for 5 days with no treatment (NT), high-dose AA (100 µg/ml) or IFN-α (1000 IU/ml). Thymidine incorporation is shown in the *y*-axis, whereas the treatment conditions are shown in the *x*-axis. ANOVA with Bonferroni post-test for multiple testing was used and the p-values are indicated by asterisks (^*^<0.05, ^**^<0.01 and ^***^<0.001). (B) NDs PBMCs (n = 3) and HAM/TSP PBMCs (n = 6) were cultured in the absence or presence of low-dose AA (10 µg/ml), high-dose AA (100 µg/ml) or IFN-α2A (1000 IU/ml) for 72 hours. Direct cell counts of viable cells were quantified by trypan blue dye exclusion. ANOVA with post-test for linear trend was used and the p-values are indicated by asterisks (^*^<0.05, ^**^<0.01 and ^***^<0.001).

Antiproliferative effects have been described both for AA as well as IFN-α. To assess the effects of AA and IFN-α on cell proliferation, we measured [^3^H]thymidine incorporation into DNA of PBMCs of seronegative normal donors (NDs) and HAM/TSP patients, an established lymphoproliferation assay. Preliminary experiments using 10–100 µg/ml of AA and 10–1000 IU/ml of IFN-α resulted in >90% cell viability at 48–72 hours of treatment, in both NDs and HAM/TSP patients (data not shown). Therefore, PBMCs were treated with fixed concentrations of 100 µg/ml of AA and 1000 IU/ml of IFN-α, both representing high doses of treatment. Spontaneous *in vitro* lymphoproliferation is a hallmark of HTLV-1 infection, triggered by *in vitro* expression of viral proteins [20]. We thus observed higher [^3^H]thymidine uptake for the untreated HAM/TSP PBMCs (3689±755.3 cpm) as compared to untreated NDs PBMCs (419±230.4 cpm, Figure 1A). High-dose AA treatment of HAM/TSP PBMCs caused a dramatic 95% decrease in spontaneous lymphoproliferation (121±51.7 cpm, Figure 1A). In contrast, IFN-α did not exert an antiproliferative effect on HAM/TSP PBMCs (3954±566.7 cpm, Figure 1A).

As a positive control, NDs PBMCs were stimulated with anti-CD3, resulting in increased [^3^H]thymidine incorporation (1926±941.3 cpm). Simultaneous addition of high-dose AA significantly reduced anti-CD3-stimulated lymphoproliferation of NDs PBMCs with 67%, when expressed as % of control (Figure S1). Similar, anti-CD3-stimulated lymphoproliferation of HAM/TSP PBMCs (4442±259.1 cpm) was also significantly reduced by 41% by simultaneous addition of high-dose AA when expressed as % of control (Figure S1). Nevertheless, the antiproliferative effect of high-dose AA was significantly higher in the absence than in the presence of anti-CD3 stimulation in HAM/TSP PBMCs, comparing mean [^3^H]thymidine uptake corresponding to high-dose AA without anti-CD3 stimulation to the mean [^3^H]thymidine uptake corresponding to high-dose AA with anti-CD3 stimulation (p = 0.0003, ANOVA, Bonferroni post-test, p<0.05), suggesting preferential inhibition of virus-*versus* TCR-induced lymphoproliferation in HAM/TSP. Moreover, as anti-CD3 stimulation did not significantly increase lymphoproliferation of HAM/TSP PBMCs (3689±755.3 cpm *vs.* 4442±259.1 cpm), inhibition of virus-induced lymphoproliferation is of more relevance than TCR-induced lymphoproliferation in HAM/TSP.

Additional experiments with direct cell counting of the number of viable cells, confirmed the contrasting effects of IFN-α and AA upon lymphoproliferation in HAM/TSP patients, as well as the absence of an antiproliferative effect in NDs. After normalization of the total viable cell counts as % of control (untreated cells), no significant effect of any drug could be observed in NDs (Figure 1B). However, in HAM/TSP patients, a dose-dependent antiproliferative effect could be confirmed (ANOVA, post-test for linear trend, p = 0.035), with IFN-α increasing the total viable cell counts, and low-dose and high-dose AA reducing the total viable cell counts in HAM/TSP PBMCs (Figure 1B). Furthermore, we also observed a significant qualitative difference in IFN-α-responders *versus* high-dose AA-responders. Using a cut-off of >25% inhibition of proliferation, none out of eight HAM/TSP patients could be defined as an IFN-α-responder, whereas six out of eight HAM/TSP patients were high-dose AA-responders (Fisher's exact test, p = 0.009), independent of the method used (thymidine incorporation and cell counting).

### AA, but not IFN-α, induces cell death in HAM/TSP PBMCs

**Figure 2:**
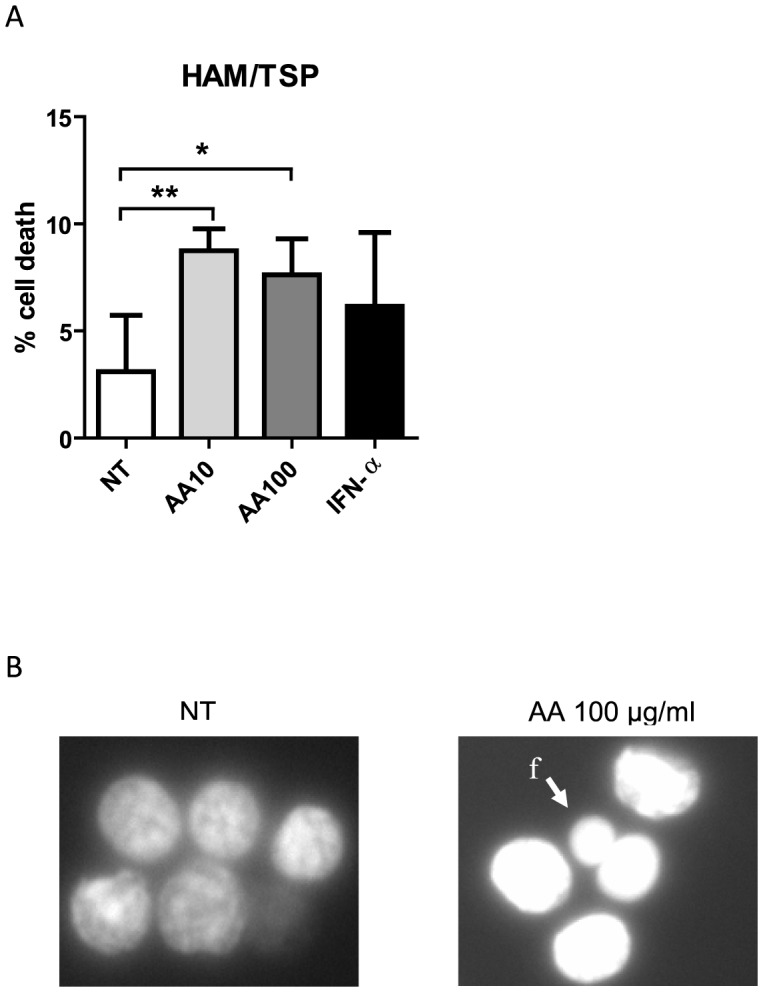
AA, but not IFN-α, induces cell death in HAM/TSP PBMCs. HAM/TSP PBMCs (n = 6) were treated for 72 hours in the absence or presence of low-dose AA (10 µg/ml), high-dose AA (100 µg/ml) or IFN-α2A (1000 IU/ml). (A) The percentage of cell death was quantified by trypan blue dye exclusion and is shown in the *y*-axis, whereas the treatment conditions are shown in the *x*-axis. The one-sample t-test p-values are indicated by asterisks (^*^<0.05, ^**^<0.01 and ^***^<0.001). (B) Fluorescence microscopy images of Hoechst 33342-stained PBMCs are shown for untreated (NT) and high-dose AA-treated PBMCs of one representative HAM/TSP patient (n = 3). Nuclear condensation and DNA fragmentation (f) was observed for AA-treated PBMCs.

Direct cell counting of both viable and dead cells by trypan blue dye exclusion, revealed modest but significant cell death induced by both low-dose AA and high-dose AA in HAM/TSP PBMCs. Hereby, low-dose AA induced 5.6±1.0% cell death and high-dose 4.5±1.6% cell death (Figure 2A). In contrast, IFN-α did not significantly induce cell death in HAM/TSP PBMCs (Figure 2A). Furthermore, fluorescence microscopy images of Hoechst 33342-stained HAM/TSP PBMCs revealed nuclear condensation and nuclear fragmentation in AA-treated PBMCs, indicating early and late stages of cell death, respectively, whereas normal nuclei were detected in untreated PBMCs, as shown in Figure 2B.

### Differential immunomodulatory effects of high-dose AA and IFN-α in HAM/TSP PBMCs

**Figure 3:**
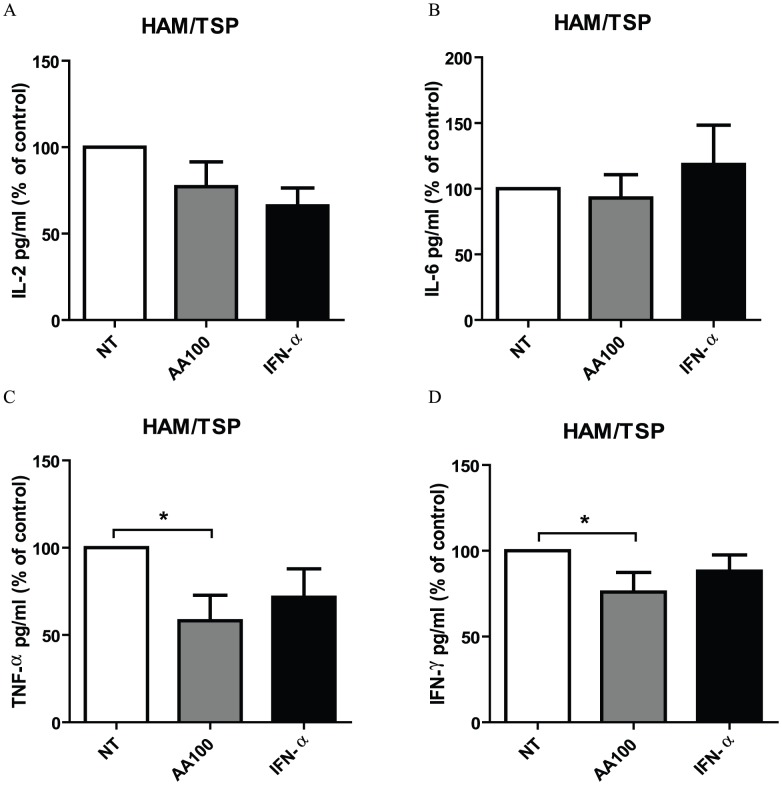
Differential immunomodulatory effects of high-dose AA and IFN-α in HAM/TSP PBMCs. Cytokine levels were quantified in cell-free supernatant of HAM/TSP PBMCs (n = 9) after 48–72 hours of treatment in the absence or presence of high-dose AA (100 µg/ml) or IFN-α (1000 IU/ml). (A) IL-2, (B) IL-6, (C) TNF-α and (D) IFN-γ levels expressed as percentage of control (untreated cells) are shown in the *y*-axis, whereas the treatment conditions are shown in the *x*-axis. ANOVA with Bonferroni post-test for multiple testing was used and the p-values are indicated by asterisks (^*^<0.05, ^**^<0.01 and ^***^<0.001).

In addition to antiproliferative effects, immunomodulatory effects have also been described both for AA as well as IFN-α. Therefore, we quantified Th1/Th2/Th17 cytokines in cell-free supernatant of unstimulated PBMCs of NDs, HTLV-1-infected asymptomatic carriers (ACs) and HAM/TSP patients, treated in the absence or presence of high-dose AA or IFN-α. None of the Th1/Th2/Th17 cytokines were detected in cell-free supernatant of NDs PBMCs, except for IL-6 which was detected in 1/7 of the NDs. A similar pattern of cytokines was observed among ACs, with very low or undetectable levels of IL-2 (2.5±1.9 pg/ml), IL-4 (0.2±0.1 pg/ml), IL-10 (0.1±0.1 pg/ml), TNF-α (20.2±19.9 pg/ml), IFN-γ (10.9±10.9 pg/ml) and IL-17 (2.2±2.2 pg/ml) and variable levels of IL-6 (2/5 ACs, 1108±1008 pg/ml). Neither high-dose AA nor IFN-α exerted an effect on Th1/Th2/Th17 cytokines levels of ACs PBMCs (Friedman test, all p>0.05). Similar to NDs and ACs, IL-4 (7.5±6.6 pg/ml), IL-10 (19.0±10.7 pg/ml) and IL-17 (3.3±1.5 pg/ml) levels were very low or undetectable in cell-free supernatant of HAM/TSP PBMCs, with no effect of IFN-α or high-dose AA (Friedman test, all p>0.05). In contrast to NDs and ACs, all of the tested pro-inflammatory cytokines could be detected in cell-free supernatant of HAM/TSP PBMCs, with strong inter-patient variability, as previously described by other groups [21]–[23]. IFN-α exerted variable effects on IL-2, IL-6, TNF-α and IFN-γ levels when expressed as % of control (Figure 3). In contrast, IFN-γ production was significantly reduced by high-dose AA with 25% in comparison to untreated cells, when expressed as % of control (Figure 3D). Furthermore, high-dose AA significantly reduced TNF-α levels with 42% in comparison to untreated cells, when expressed as % of control (Figure 3C). Variable effects of high-dose AA were observed on IL-2 and IL-6 levels (Figure 3A, 3B, respectively).

Th1/Th2/Th17 cytokines quantification was carried out in different laboratories (Peru *versus* Brazil), explaining the difference in experimental conditions (48 h *vs.* 72 h). In order to allow comparison of both time points, we quantified cytokine levels in cell-free supernatant of NDs, ACs and HAM/TSP PBMCs at 24, 48 and 72 hours of stimulation. We observed gradual accumulation of cytokines in the supernatant of PBMCs (HAM>ACs, absent in NDs), but with a similar degree of drug inhibition at 48 *vs.* 72 hours (data not shown). For all cytokines tested, the % inhibition by either high-dose AA or IFN-α was not significantly different at 48 and 72 hours (ANOVA with Bonferroni post-test, all comparisons, p>0.05), thus allowing us to combine these results for a pooled analysis. Altogether, high-dose AA significantly reduced IFN-γ and TNF-α pro-inflammatory cytokine levels, whereas IFN-α exerted variable effects, demonstrating differential immunomodulatory effects of high-dose AA in comparison to IFN-α.

### Variable effects of both high-dose AA and IFN-α upon p19 levels in HAM/TSP PBMCs

IFN-α has been reported to inhibit viral assembly and release in HTLV-1-infected cell lines through the prevention of Gag protein interaction with lipid rafts [24]. Therefore, we quantified HTLV-1 p19 protein levels in cell-free supernatant of PBMCs of NDs (as a negative control), ACs and HAM/TSP patients, treated in the absence or presence of IFN-α or high-dose AA. HTLV-1 p19 levels were undetectable in cell-free supernatant of both NDs PBMCs and ACs PBMCs. In addition, non-significant effects of IFN-α (66% decrease) and high-dose AA (9% increase) were observed upon HTLV-1 p19 levels in cell-free supernatant of HAM/TSP PBMCs, due to strong inter-patient variability (Friedman test, p>0.05, data not shown).

We thus hypothesized that the significant antiproliferative and immunomodulatory effects of high-dose AA might be due to elimination of HTLV-1-infected cells, rather than through an antiviral effect. Therefore, we investigated the possible effects of AA and IFN-α treatment on HTLV-1-infected CD4+ T cell lines, MT-2 and MT-4, as HTLV-1 has a preferential tropism for CD4+ T cells *in vivo* [25].

### AA treatment dose-dependently induces cell death in HTLV-1-infected cell lines

**Figure 4:**
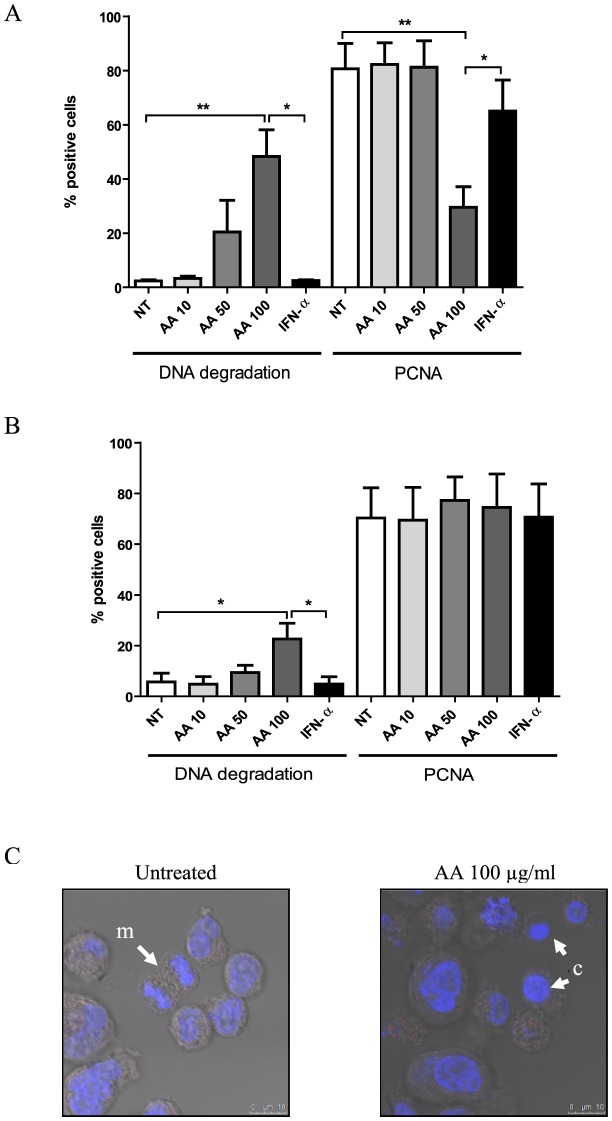
AA treatment dose-dependently induces cell death in HTLV-1-infected cell lines. HTLV-1-infected CD4+ T-cell lines were cultured for 48 hours with no treatment (NT), low-, intermediate-, high-dose AA (10, 50, 100 µg/ml) or IFN-α (1000 IU/ml). Flow cytometric quantification of cells with DNA degradation (Hoechst 33342-positive subdiploid cells) and proliferating cell nuclear antigen (PCNA)-positive cells are shown in the *y*-axis for both (A) MT-4 (n = 4) as well as (B) MT-2 cells (n = 3), whereas the treatment conditions are shown in the *x*-axis. ANOVA with Bonferroni post-test for multiple testing was used and the p-values are indicated by asterisks (^*^<0.05, ^**^<0.01 and ^***^<0.001). (C) Confocal microscopy images of Hoechst 33342-stained MT-2 cells are shown for untreated and high-dose AA-treated MT-2 cells. Nuclear condensation (c) was observed for the AA-treated cells, whereas normal nuclei and mitosis (m) were observed for the untreated cells.

HTLV-1-infected cell lines were cultured for 48 hours in the absence or presence of low-, intermediate- and high-dose AA or IFN-α. Flow cytometric analysis of MT-4 cells revealed a significant dose-dependent cell death-inducing effect of AA (ANOVA, post-test for linear trend, p = 0.0009), being maximal at high-dose with a 45.9±9.9% increase in DNA degradation (Figure 4A). Moreover, high-dose AA induced a 51.1±7.6% decrease in PCNA expression of the remaining viable HTLV-1-infected MT-4 cells, suggesting an additional antiproliferative effect of high-dose AA (Figure 4A). Flow cytometric analysis of MT-2 cells revealed no antiproliferative, yet again dose-dependent cell death-inducing effects of AA (ANOVA, post-test for linear trend, p = 0.0058), inducing a less pronounced but significant 16.9±6.2% increase in DNA degradation (Figure 4B). Furthermore, confocal microscopy images of Hoechst-stained HTLV-1-infected cells revealed nuclear condensation for the AA-treated cells, whereas normal nuclei and mitosis were observed for the untreated cells (Figure 4C), confirming cell death-inducing and antiproliferative effects of AA. Nevertheless, flow cytometric analysis of both MT-2 and MT-4 cells revealed no effect of AA on active-caspase 3 expression (data not shown). Of note, AA treatment did not alter the pH of the culture medium (supplemented with 10 mM HEPES), even at high-dose, thereby excluding the possibility that cell death might be caused by mere acidification. In contrast, IFN-α had no significant cell death-inducing nor antiproliferative effects in MT-4 and MT-2 cells (Figure 4A and 4B). In addition, as AA has profound antioxidant properties, we evaluated the effect of NAC (10 mM), another antioxidant, on MT-2 and MT-4 cells. Flow cytometric analysis revealed no effect of NAC on cell death nor proliferation after 48 hours (data not shown). Furthermore, at the same concentrations tested, neither AA nor IFN-α exerted effects on cell death nor proliferation of the HTLV-negative Jurkat T-cell line (data not shown).

### Contrasting effects of high-dose AA and IFN-α on cytokine levels in HTLV-1-infected cell lines

**Figure 5:**
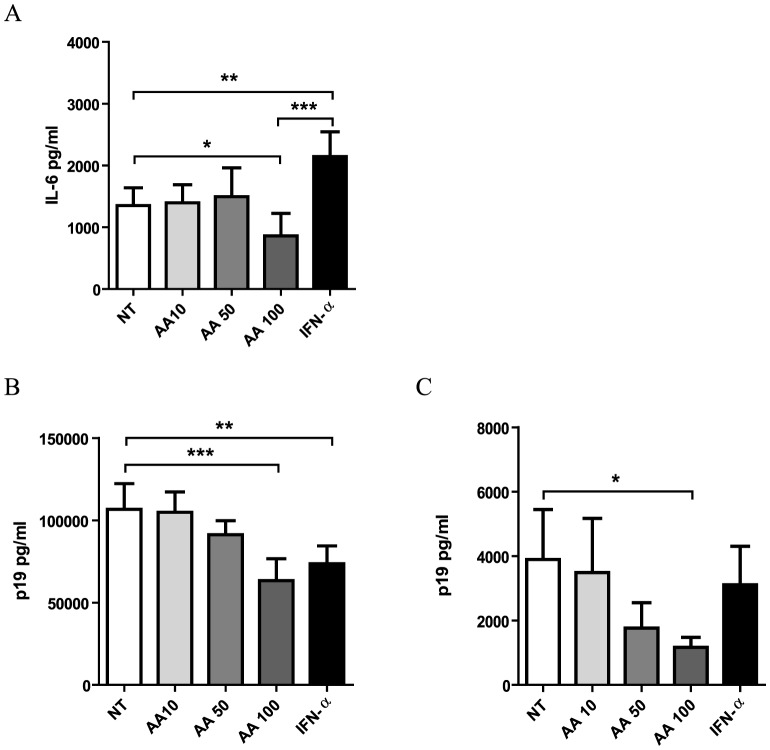
High-dose AA treatment decreases IL-6 and HTLV-1 p19 levels in HTLV-1-infected cell line supernatant. HTLV-1-infected CD4+ T-cell lines were cultured for 48 hours with no treatment (NT), low-, intermediate-, high-dose AA (10, 50, 100 µg/ml) or IFN-α (n = 4). (A) IL-6 levels were quantified in cell-free supernatant of MT-2 cells and shown in the *y*-axis. HTLV-1 p19 concentration (pg/ml) was determined in cell-free supernatant of (B) MT-2 and (C) MT-4 cells and shown in the *y*-axis. The treatment conditions are shown in the *x*-axis. ANOVA with Bonferroni post-test for multiple testing was used and the p-values are indicated by asterisks (^*^<0.05, ^**^<0.01 and ^***^<0.001).

In parallel with patient samples, we quantified Th1/Th2/Th17 cytokines in cell-free supernatant of MT-2 and MT-4 cells. Surprisingly, no cytokines could be detected in cell-free supernatant of MT-4 cells, whereas MT-2 cells only secreted high levels of IL-6 and intermediate levels of IL-10. High-dose AA significantly reduced IL-6 secretion with 37% in comparison to untreated cells, whereas IFN-α significantly increased IL-6 secretion (Figure 5A). In contrast, intermediate-dose and low-dose AA exerted no effect on IL-6 levels. Neither high-, intermediate- or low-dose AA, nor IFN-α exerted an effect on IL-10 levels (data not shown). We thus observed contrasting immunomodulatory effects of both drugs, with IFN-α up-regulating and high-dose AA down-regulating IL-6 secretion in MT-2 cells.

### Both high-dose AA and IFN-α decrease p19 levels in cell-free supernatant of HTLV-1-infected cell lines

In parallel with patient samples, we quantified p19 levels in cell-free supernatant of MT-2 and MT-4 cells. AA treatment dose-dependently reduced p19 levels in cell-free supernatant of both MT-2 and MT-4 cells (ANOVA, post-test for linear trend, p = 0.0001, p = 0.0083, Figure 5B and 5C, respectively). Maximum inhibition of p19 secretion was observed for high-dose AA with 41% reduction for MT-2 and 70% reduction for MT-4 cells, in comparison to untreated cells. IFN-α treatment reduced p19 concentration by 31% for MT-2 and by 20% for MT-4 cells, although without reaching statistical significance in MT-4 cells.

### Gene expression profiling of IFN-α- and AA-treated MT-2 cells

AA has been reported to affect multiple pathways including antioxidant and immunity pathways [26]. In order to integrate our data on cell death, proliferation and viral expression and explore the molecular mechanism(s) of action of AA in the context of HTLV-1 infection, gene expression profiling was performed. We selected MT-2 cells at 48 hours of treatment for RNA extraction in order to ensure a maximal biological effect while still maintaining cell viability (±80%), since cell death was too advanced in MT-4 cells (±45%), which resulted in poor RNA yield. Whole humane genome microarray (Affymetrix Human Gene 1.0 ST Array) analysis was performed with triplicate samples for MT-2 cells. Gene-expression profiling of MT-2 revealed 142 genes significantly regulated by high-dose AA, of which 48 were down- and 94 were up-regulated. Approximately 37% (52/142) of high-dose AA-regulated genes were unknown. The top 20 of the most significant known genes, down- and up-regulated by high-dose AA, are shown in Table 2. Intermediate-dose AA significantly regulated 17 genes, of which 15 were down- and 2 were up-regulated. Low-dose AA significantly regulated 12 genes, of which 9 were down- and 3 were up-regulated. In comparison to high-dose AA, five common regulated genes were identified for intermediate-dose AA, including microRNA 155. For low-dose AA, only transmembrane channel-like 7 was common with high-dose AA-regulated genes. Moreover, the number of genes regulated by AA treatment was proportional with the used doses, confirming a dose-dependent effect. In parallel, gene-expression profiling of MT-2 revealed 93 genes significantly regulated by IFN-α, of which 3 were down- and 90 were up-regulated. The down-regulated genes and the top 20 of the most significant up-regulated genes are shown in Table 3. In comparison to AA treatment, only a minority of IFN-α-regulated genes appeared to be unknown (±7% or 6/93). Known IFN-α-regulated genes included classical interferon-stimulated genes (ISGs) such as myxovirus resistance (Mx) and 2′,5′-oligoadenylate synthetase (OAS) genes. Based on the AA- and IFN-α-regulated genes, identified via microarray analysis, Ingenuity Pathway Analysis (IPA) was performed in order to identify pathways affected by AA and IFN-α in HTLV-1-infected MT-2 cells.

**Table 2:** Overview of the top 20 of the most significant down- and up-regulated genes by high-dose AA (100 µg/ml) in MT-2 cells.

Top 20 of down-regulated genes		
Gene Symbol	Gene Description	Chromosome
BEST1	bestrophin 1	chr11
INHBE	inhibin, beta E	chr12
MIR155	microRNA 155	chr21
SLC7A11	solute carrier family 7, member 11	chr4
TRIM43	tripartite motif-containing 43	chr2
SNORD116-29	small nucleolar RNA, C/D box 116-29	chr15
SULF1	sulfatase 1	chr8
NRXN3	neurexin 3	chr14
C1orf113	chromosome 1 open reading frame 113	chr1
CHAC1	ChaC, cation transport regulator homolog 1 (E. coli)	chr15
SNORD116-13	small nucleolar RNA, C/D box 116-13	chr15
MINA	MYC induced nuclear antigen	chr3
SNORD116-27	small nucleolar RNA, C/D box 116-27	chr15
IGSF9B	immunoglobulin superfamily, member 9B	chr11
ITK	IL2-inducible T-cell kinase	chr5
ITGBL1	integrin, beta-like 1 (with EGF-like repeat domains)	chr13
SLC6A9	solute carrier family 6 (neurotransmitter transporter, glycine), member 9	chr1
PGBD1	piggyBac transposable element derived 1	chr6
GALNT4	UDP-N-acetyl-alpha-D-galactosamine (GalNAc-T4)	chr12
CD40	CD40 molecule, TNF receptor superfamily member 5	chr20

Top 20 of up-regulated genes		
Gene Symbol	Gene Description	Chromosome
IL23R	interleukin 23 receptor	chr1
CD69	CD69 molecule	chr12
GIMAP5	GTPase, IMAP family member 5	chr7
TLL1	tolloid-like 1	chr4
TLE4	transducin-like enhancer of split 4	chr9
KYNU	kynureninase (L-kynurenine hydrolase)	chr2
PROL1	proline rich, lacrimal 1	chr4
MGP	matrix Gla protein	chr12
GNG4	guanine nucleotide binding protein (G protein), gamma 4	chr1
TOB2	transducer of ERBB2, 2	chr5
TOB2	transducer of ERBB2, 2	chrM
PRSS55	protease, serine, 55	chr8
TAS2R9	taste receptor, type 2, member 9	chr12
PCDHB3	protocadherin beta 3	chr5
TMC7	transmembrane channel-like 7	chr16
LOC100510740	ATP synthase subunit f, mitochondrial-like	chr17
PLAC1L	placenta-specific 1-like	chr11
C6orf94	chromosome 6 open reading frame 94	chr6
HIST1H1A	histone cluster 1, H1a	chr6
RPS24	ribosomal protein S24	chr10

**Table 3:** Overview of the significant down-regulated genes and the top 20 of the most significant up-regulated genes by IFN-α in MT-2 cells.

Down-regulated genes		
Gene Symbol	Gene Description	Chromosome
PGBD1	piggyBac transposable element derived 1	chr6
HIST1H3B	histone cluster 1, H3b	chr6

Top 20 of up-regulated genes		
Gene Symbol	Gene Description	Chromosome
OAS2	2′-5′-oligoadenylate synthetase 2, 69/71 kDa	chr12
OAS1	2′,5′-oligoadenylate synthetase 1, 40/46 kDa	chr12
IFITM1	interferon induced transmembrane protein 1 (9–27)	chr11
IFI44L	interferon-induced protein 44-like	chr1
MX1	myxovirus (influenza virus) resistance 1	chr21
DDX60	DEAD (Asp-Glu-Ala-Asp) box polypeptide 60	chr4
OAS3	2′-5′-oligoadenylate synthetase 3, 100 kDa	chr12
IFI44	interferon-induced protein 44	chr1
IFIT5	interferon-induced protein with tetratricopeptide repeats 5	chr10
PLSCR1	phospholipid scramblase 1	chr3
IFI6	interferon, alpha-inducible protein 6	chr1
SP100	SP100 nuclear antigen	chr2
DDX58	DEAD (Asp-Glu-Ala-Asp) box polypeptide 58	chr9
EIF2AK2	eukaryotic translation initiation factor 2-alpha kinase 2	chr2
PARP14	poly (ADP-ribose) polymerase family, member 14	chr3
XAF1	XIAP associated factor 1	chr17
IFI35	interferon-induced protein 35	chr17
DHX58	DEXH (Asp-Glu-X-His) box polypeptide 58	chr17
USP18	ubiquitin specific peptidase 18	chr22
TRIM25	tripartite motif-containing 25	chr17

### Pathway analysis in AA-treated MT-2 cells confirms the role of AA-regulated cell death

**Figure 6:**
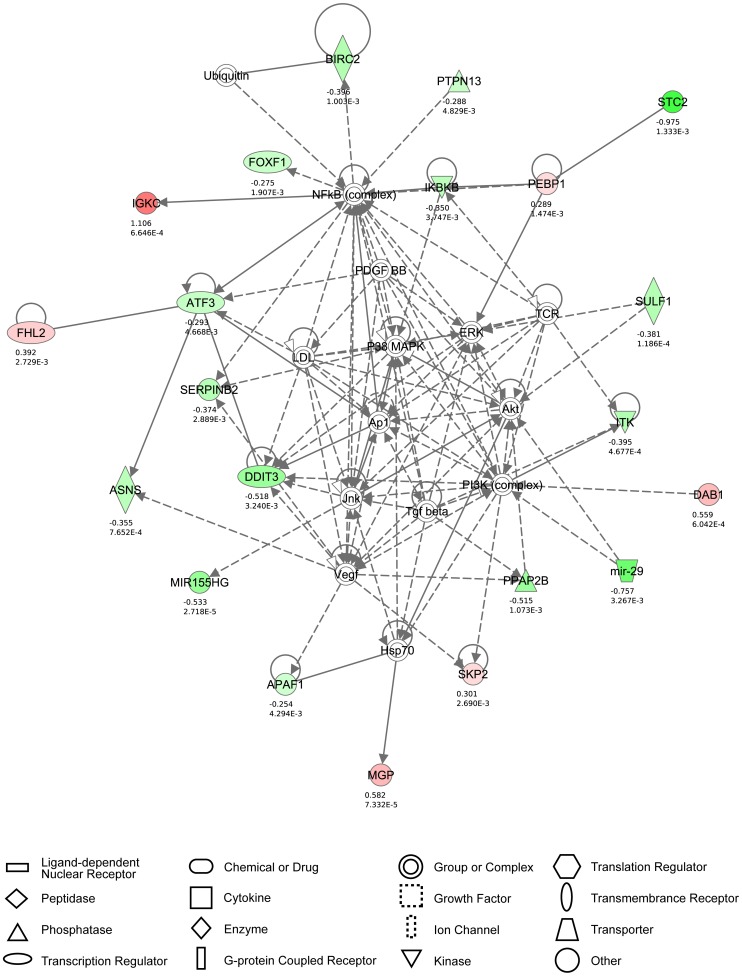
Cell death is the principal high-dose AA-modulated molecular network in MT-2 cells. The networks were generated through the use of IPA (Ingenuity® Systems, www.ingenuity.com), based on genes regulated by high-dose AA treatment in MT-2 cells (after 48 hours of culture). The principal molecular network is graphically displayed, identified as cell death. The red and green colour indicate up- or down-regulation, respectively, with the intensity indicating the degree of the gene transcription change. The log_2_ fold-change values and p-values are indicated below each molecule. Network shapes are represented in the legend. Full lines represent direct interactions, while dashed lines indirect interactions.

Genes up- and down-regulated by both AA and IFN-α were sorted into molecular gene networks, of which significantly overrepresented networks were identified. In the case of IFN-α treatment, eight significantly modulated molecular networks could be identified, of which the principal network contained 27 genes, all up-regulated, and represented antimicrobial response. In the case of high-dose AA treatment, 25 significantly modulated molecular networks could be identified, of which the principal network contained 21 genes, both up- and down-regulated, and represented cell death (Figure 6). Strikingly, this network comprised Akt, PI3K, ERK, p38 MAP kinase, Vegf and Jnk, all of which have been described to play pivotal roles in HTLV-1-regulated proliferation and/or cellular activation [27]–[31]. In the case of intermediate-dose AA, twelve significantly modulated molecular networks could be identified, of which the principal network contained 16 genes, both up- and down-regulated, and also represented cell death. In the case of low-dose AA, eleven significantly modulated molecular networks could be identified, of which the principal network contained 10 genes, both up- and down-regulated, and again represented cell death. Taken together, the most relevant pathway dose-dependently modulated by AA treatment was related to cell death, whereas IFN-α up-regulated antiviral pathways.

## Discussion

The goal of our study was not to offer a rationale for or to recommend the use of AA *in vivo*. Rather since high-dose AA is already administered *in vivo*, and both IFN-α and high-dose AA have similar success rates *in vivo* [8], our study was intended to perform the first comparative analysis of their *ex vivo* and *in vitro* molecular and cellular mechanisms of action. In this study, we reveal significant and superior antiproliferative, cell death-inducing and immunomodulatory effects of high-dose AA compared to IFN-α treatment, as demonstrated *ex vivo* in primary HAM/TSP PBMCs.

In spite of the pronounced antiproliferative effects of high-dose AA in HAM/TSP PBMCs, treatment of NDs PBMCs also revealed antiproliferative effects of high-dose AA, unrelated to HTLV-1, indicating broad antiproliferative effects. Nevertheless, we observed preferential inhibition of virus-*versus* TCR-induced lymphoproliferation in HAM/TSP, in keeping with its excellent safety profile and mild side effects *in vivo* [8]. A distinct pattern of Th1/Th2/Th17 cytokines was observed for NDs and ACs in comparison with HAM/TSP patients, detected in cell-free supernatant of PBMCs. Whereas a minority of NDs and ACs secreted IL-6 only, the majority of HAM/TSP patients produced IL-2, IL-6, TNF-α and IFN-γ pro-inflammatory cytokines. Although IFN-α exerted variable effects on pro-inflammatory cytokine levels, it non-significantly reduced IL-2 levels in HAM/TSP patients. However, IFN-α was unable to inhibit lymphoproliferation, in agreement with the previously reported modest role of IL-2 in spontaneous lymphoproliferation [32]–[34]. Furthermore, high-dose AA, but not IFN-α, significantly reduced IFN-γ and TNF-α levels of HAM/TSP PBMCs. Although the effects of IFN-α and high-dose AA on cytokine levels of HAM/TSP PBMCs were not significantly different, we suggest that high-dose AA has differential and superior immunomodulatory effects over IFN-α in HAM/TSP PBMCs, given the exacerbated *in vitro* production of primarily IFN-γ and TNF-α by PBMCs from HAM/TSP patients and their high *in vivo* levels in cerebrospinal fluid and spinal cord lesions of HAM/TSP patients [22], [35]–[39]. Interestingly, the immunomodulatory effects upon cytokine levels, the induction of cell death or the antiproliferative response of either AA or IFN-α were not intercorrelated, supporting the differential pathways used by both drugs revealed by microarray data. In contrast, no significant effect of IFN-α nor high-dose AA was observed on HTLV-1 p19 levels in cell-free supernatant of HAM/TSP PBMCs, due to strong inter-patient variability. Of note, the antiproliferative or immunomodulatory effects of both drugs were also independent of the proviral load of HAM/TSP patients (p>0.3 for all comparisons). Given that certain HAM/TSP patients included in our study were already treated with high-dose AA combined with prednisone, we also recruited additional untreated Brazilian HAM/TSP patients, as well as Peruvian patients who were being treated symptomatically (baclofen) or had received antiretrovirals (AZT and 3TC). No correlation was observed between *ex vivo* drug response and EDSS or disease duration. Therefore, the higher EDSS in some of our patients merely reflects more aggressive disease and rapid progression in Brazilian and Peruvian cohorts, as previously demonstrated [40], [41], as compared to for example the Japanese cohorts.

Due to the strong variability as well as cellular heterogeneity in HAM/TSP patient samples, we aimed to confirm our *ex vivo* findings in HTLV-1-infected CD4+ T-cell lines *in vitro*. In agreement with our HAM/TSP data, we were able to confirm the absence of antiproliferative and anti-inflammatory effects of IFN-α in HTLV-1-infected cell lines. In addition, we confirmed the previously described posttranscriptional inhibition of HTLV-1 p19 secretion by IFN-α [24] in HTLV-1-infected cell lines, without any effect of IFN-α on cell death. Furthermore, we were able to confirm the antiproliferative, cell death-inducing and immunomodulatory effects of high-dose AA in both HTLV-1-infected cell lines, although MT-4 cells appear to be more sensitive to AA treatment than MT-2 cells. Whereas AA dose-dependently induced cell death in HTLV-1-infected cell lines, only high-dose AA exerted antiproliferative and immunomodulatory effects. We speculate that in analogy with the *in vivo* situation in HAM/TSP patients, only high-dose AA is sufficient to induce significant effects. We hypothesize that the cell death-inducing and immunomodulatory effects of high-dose AA in HTLV-1-infected cell lines were most probably a direct consequence of programmed cell death, with morphological evidence of apoptosis. However, active-caspase 3 activation was not detected, suggesting that the effect of high-dose AA on DNA degradation and cell death might not be mediated by classical, caspase-dependent apoptosis. Confocal microscopy images confirmed extensive cell death with nuclear condensation in cell lines, but without massive accumulation of classical apoptotic bodies. In addition, IPA identified cell death-associated networks rather than classical caspase-dependent apoptosis, suggesting other types of cell death, such as necroptosis, caspase-independent apoptosis and/or mitotic catastrophe, should be considered as well. Although reactive oxygen species, as a major player in apoptotic cell death, are an obvious target of AA, genes or signaling pathways related to oxidative stress were not significantly up-regulated by AA treatment. In addition, treatment of HTLV-1-infected cell lines with *N*-acetylcysteine showed no effect on cell death or proliferation, suggesting that the inhibitory effects of AA are unrelated to its antioxidant properties. Another possible explanation for the anti-HTLV-1 effect of AA, might be through the binding of its oxidized form, dehydroascorbic acid (DHA), to the ubiquitous HTLV-1 receptor GLUT-1 [42] and thereby blocking cell-to-cell viral spread or through interactions of DHA with cellular pathways involved in cell proliferation or survival, such as NF-κB. However, in contrast with AA, DHA did not induce cell death or DNA degradation at either low- or high-dose in HTLV-1-infected cell lines (data not shown). In addition, significantly high-dose AA-modulated carbohydrate metabolism and starch and sucrose metabolism canonical pathway, did not include GLUT-1 (or any related glucose-transporter). Therefore, pooled analysis by microarray and IPA indicate that the cell death-inducing effects of high-dose AA treatment in MT-2 cells, are likely mediated by genes such as ATF3, IKBKB, FOXF1, PTPN13, SERPINB2 or MIR155 (Figure 6). Of those, IκB has been previously associated, whereas the others represent novel molecular targets in HAM/TSP. ATF3 has been shown to directly bind HBZ [43], the HTLV-1 antisense transcript known to induce proliferation of HTLV-1-infected cells [44], [45] and to positively correlate with HAM/TSP disease severity [46]. miR-155 is one of the few well-studied microRNAs that has been linked to immune system function and oncogenesis [47]–[49]. Through the promotion of the development of inflammatory T cells, including the IFN-γ-producing Th1, and the IL-17-producing Th17 cell subsets, and T cell-dependent tissue inflammation, miR-155 could be a key player in various autoimmune diseases [50], [51]. In both brain lesions as well as PBMCs of multiple sclerosis patients, miR-155 has been shown to be up-regulated [52], [53]. In addition, miR-155 has also been shown to function as a positive regulator of IFN-γ production in natural killer cells [54]. In HTLV-1-transformed cells, miR-155 has been reported to be up-regulated when compared to HTLV-negative control cells [55], [56]. Given that HAM/TSP is characterized by a vigorous immune response to HTLV-1 with an exacerbated *in vivo* production of IFN-γ, dysregulation of miR-155 could contribute to the development of HAM/TSP. Our results, revealing high-dose AA-induced down-regulation of miR-155 in MT-2 cells, suggest that this microRNA could represent a novel therapeutic target in HAM/TSP. In parallel, microarray and IPA analysis confirmed IFN-α-activated signaling pathways, resulting in the induction of several known antiviral genes such as OAS, Mx, IFI35 and IFITM1. Nevertheless, large clinical studies are necessary to elucidate the relevance of these IFN-α- and AA-regulated pathways in HAM/TSP *in vivo*. Moreover, as IFN-α has a higher cost price and more severe side effects in comparison to high-dose AA treatment, the therapeutic potential of high-dose AA should be further explored, in parallel with widely used treatments such as corticosteroids and IFN-α, in future clinical studies with a biomarker discovery design. Considering the differential *ex vivo* and *in vitro* effects of AA and IFN-α, as demonstrated in this study, their modest *in vivo* effectiveness might be increased if host or viral biomarkers are identified that reliably predict treatment outcome.

In conclusion, high-dose AA treatment has superior *ex vivo* and *in vitro* cell death-inducing, antiproliferative and immunomodulatory anti-HTLV-1 effects, as compared to IFN-α. However, differential pathway activation by both drugs opens up avenues for targeted treatment in specific patient subsets. Our findings reveal molecular mechanisms of action as well as candidate biomarkers for both IFN-α and high-dose ascorbic acid therapy and provide a rational basis for their use in HAM/TSP treatment.

## Supporting Information

Figure S1 **High-dose AA exerts broad antiproliferative effects in both NDs and HAM/TSP PBMCs.** NDs PBMCs (n = 4) and HAM/TSP PBMCs (n = 3) were treated for 5 days with anti-CD3 monoclonal antibody (0.2 µg/ml) in the absence or presence of high-dose AA (100 µg/ml), performed in triplicate. Thymidine incorporation is expressed as percent of control (untreated cells) and shown in the *y*-axis, whereas the treatment conditions are shown in the *x*-axis. The one-sample t-test p-values are indicated by asterisks (^*^<0.05, ^**^<0.01 and ^***^<0.001).
(EPS)
